# A Rare Case of Autoimmune Enteropathy After Thymectomy

**DOI:** 10.7759/cureus.76020

**Published:** 2024-12-19

**Authors:** Arti Patel, Paul Formaker, Jonathan Abaya Ghazaleh, Adewale Ajumobi

**Affiliations:** 1 Internal Medicine, Eisenhower Health, Rancho Mirage, USA; 2 Graduate Medical Education, Eisenhower Health, Rancho Mirage, USA; 3 Gastroenterology, Eisenhower Health, Rancho Mirage, USA

**Keywords:** anti-enterocyte antibodies, anti-goblet cell antibodies, autoimmune enteropathy, bilateral pleural effusion, hypoalbuminemia, persistent diarrhea, thymectomy

## Abstract

Autoimmune enteropathy (AIE) is a rare cause of chronic diarrhea associated with autoantibodies and susceptibility to other autoimmune diseases, such as rheumatoid arthritis, diabetes, autoimmune hemolytic anemia, and atopic dermatitis. While it is more common in children, the prevalence of AIE in adults is increasing. Due to the nonspecific nature of its presenting symptoms and the lack of consistent findings, AIE can be challenging to diagnose. Here, we present a 66-year-old male patient who presented to the emergency department with diarrhea and recurrent bilateral pleural effusions two months after thymoma resection and was eventually diagnosed with AIE. The evaluation revealed blunting of small intestinal villi on biopsy, an IgG staining pattern indicative of circulating anti-enterocyte antibodies, as well as the presence of serum anti-enterocyte and anti-goblet cell antibodies, establishing the diagnosis of AIE.

## Introduction

Autoimmune enteropathy (AIE) is an uncommon cause of chronic diarrhea associated with autoantibodies and linked to other autoimmune conditions. The incidence of AIE in children between the ages of zero to 16 years was reported to be 0.06 per 100,000 children. However, no data is available regarding the incidence in adults, as described in a meta-analysis in 2019 [1]. The proposed major criteria for diagnosis of AIE include adult-onset chronic diarrhea lasting greater than six weeks, malabsorption, complete or partial villous blunting on small intestinal biopsies, deep crypt lymphocytosis, increased crypt apoptotic bodies, and minimal intraepithelial lymphocytosis [1,2]. Moreover, other causes of villous atrophy, such as celiac disease, refractory sprue, and intestinal lymphoma must be excluded [2]. Though anti-enterocyte and anti-goblet cell autoantibodies support the diagnosis, they are not necessary for it [2].

The pathophysiology of AIE is not fully understood, but current studies on immune dysregulation, polyendocrinopathy, and enteropathy X-linked (IPEX) syndrome (a rare subgroup of AIE observed in children) demonstrate dysregulation in T-cell homeostasis that leads to immune hyperactivity [3,4]. The cluster of differentiation CD25+CD4+ regulatory T cells, responsible for maintaining immunological self-tolerance and downregulating immune responses, express forkhead box protein P3 (FOXP3), a transcription regulator that encodes a transcription-repressor protein known as scurfin [3,4]. Scurfin is understood to be involved in the regulation and suppression of T-cell activation [5]. Thus, mutations in the FOXP3 gene lead to hyperactivation of the T cells observed in patients with the IPEX syndrome [4,6]. The pathophysiology of AIE is assumed to be similar [7], but further research is needed. We discuss a case of a 66-year-old male patient who presented with diarrhea and recurrent bilateral pleural effusions two months post-thymectomy and was ultimately diagnosed with AIE.

## Case presentation

Our patient was a 66-year-old male patient with a past medical history of thymoma, World Health Organization (WHO) AB Masaoka stage IV, diagnosed two months prior to presentation. He subsequently underwent excision with clear margins, anterior pericardiotomy, and mediastinal nodal dissection. He presented to the emergency department with non-bloody diarrhea, intractable nausea and vomiting, and minimal oral intake for four weeks, without any change in weight. His family history was noncontributory.

At the time of the thymoma diagnosis, he had presented with pericardial effusion and bilateral pleural effusions, requiring bilateral thoracenteses and eventual left PleurX catheter (Becton Dickinson, New Jersey, US) placement. Post-thymectomy, the patient was followed up by cardiothoracic surgery and pulmonology services, however, he presented to the hospital due to worsening gastrointestinal symptoms. The plan was also to place a right PleurX catheter due to the recurrence of the pleural effusions.

During this admission, it was noted that he was borderline hypotensive with mean arterial pressures in the high 60s and hypoxic to 91%, requiring two liters of supplemental oxygenation. The patient's physical examination was notable for diminished breath sounds at the lung bases bilaterally, bilateral lower extremity 2+ pitting edema, and anasarca. His laboratory values were significant for decreased total protein at 5.4 g/dL (reference range 6.4-8.9 g/dL), hypoalbuminemia at 2.8 g/dL (reference range 3.5-5.7 g/dL), leukocytosis at 13.3 K/µL (reference range 3.8-10.8 K/µL), mildly elevated aspartate aminotransferase (AST) at 43 U/L (reference range 13-39 U/L), and elevated fecal calprotectin at 5120 mcg/g (reference range <50 mcg/g). Thyroid stimulating hormone and cortisol were also within normal limits. The remaining laboratory values in the comprehensive metabolic panel and complete blood count were unremarkable. The gastrointestinal panel was negative for bacterial and viral gastroenteritis (Table 1).

**Table 1:** Gastrointestinal panel was negative for bacterial or viral gastroenteritis E.coli, Escherichia coli; LT, heat labile; ST, heat stable; Adenovirus F40/41, serotypes 40 and 41 of adenovirus.

Bacteria/viruses	Reference range (Not detected = negative)
Campylobacter coli/jejuni/upsaliensis	Not detected
Clostridium difficile toxin A/B gene	Not detected
Plesiomonas shigelloides	Not detected
Salmonella enterica/bongori	Not detected
Vibrio	Not detected
Vibrio cholera/parahaemolyticus/vulnificus	Not detected
Yersinia enterocolitica	Not detected
Enteroaggregative E. coli (EAEC)	Not detected
Enteropathogenic E. coli (EPEC)	Not detected
Enterotoxigenic E. coli (ETEC) LT/ST	Not detected
Shiga-like toxin-producing E. coli (STEC)	Not detected
Shigella/Enteroinvasive E. coli (EIEC)	Not detected
Cryptosporidium	Not detected
Cyclospora caveanesis	Not detected
Entamoeba histolytica	Not detected
Giardia lamblia	Not detected
Adenovirus F40/41	Not detected
Astrovirus	Not detected
Norovirus genogroups I/II	Not detected
Rotavirus A	Not detected
Sapovirus genogroups I/II/III/IV	Not detected

**Figure 1:**
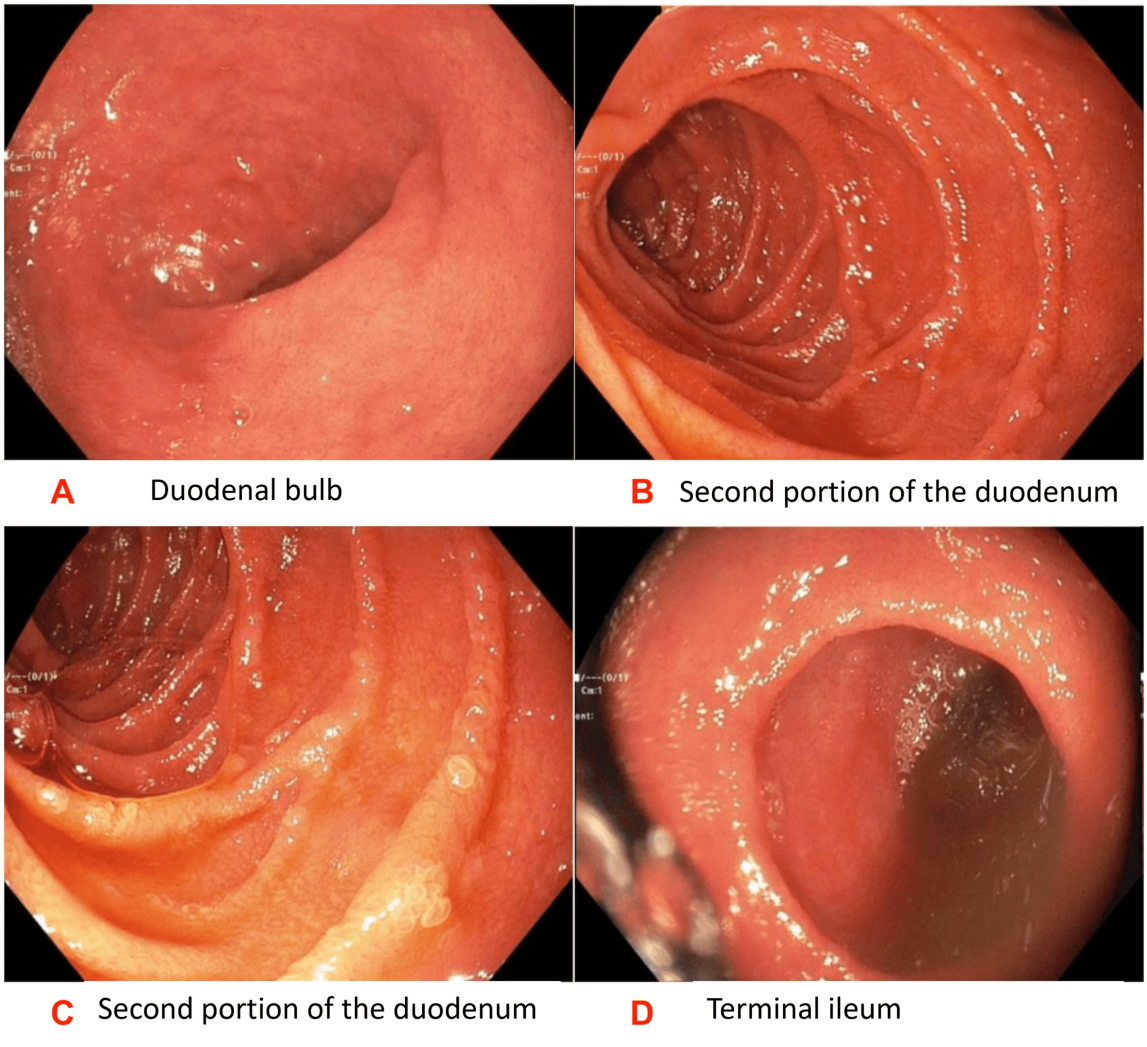
Grossly unremarkable esophagogastroduodenoscopy (EGD) and colonoscopy 1A: Duodenal bulb on EGD; 1B and 1C: Second portion of the duodenum on EGD; 1D: Terminal ileum on colonoscopy.

Computed tomography (CT) of the abdomen and pelvis was significant for enterocolitis for which ceftriaxone and metronidazole were started. However, antibiotics were eventually discontinued in a few days due to a lack of clinical improvement. Gastroenterology service was consulted and the patient underwent esophagogastroduodenoscopy (EGD). Although the EGD was grossly unremarkable (Figures 1A-1C), duodenal biopsies were significant for occasionally partially-blunted villi with a slight increase in lymphocytes within the interstitium. A trial of antidiarrheals was initiated, however, due to persistence of diarrhea, colonoscopy was performed. It was also grossly unremarkable (Figure 1D).

Biopsies from the terminal ileum were significant for active ulcerative ileitis with inflamed granulation tissue and adjacent disrupted, focally partially blunted villi. Immunofluorescence was performed on sections of normal human small bowel using the patient's serum as a first stage and fluorescein isothiocyanate (FITC)-conjugated IgG, IgA, and IgM on sequential slides as a second stage. For IgG, positive linear staining of the apical enterocyte membranes was noted. This IgG staining pattern is indicative of circulating anti-enterocyte antibodies and, in the appreciated clinical setting, is consistent with AIE [8]. Autoimmune workup was positive for antinuclear antibodies (ANA) with titers 1:80 (reference value <1:40) and elevated C-reactive protein (CRP) at 6.8 mg/dL (reference value <1.0 mg/dL). Anti-tissue transglutaminase and anti-deamidated gliadin IgA antibodies were negative, however, anti-enterocyte and anti-goblet cell antibodies were positive. Other infectious disease workups were negative. It is unlikely that the patient’s presentation was due to renal disease because the urine protein-to-creatinine ratio was normal (reference value <0.2), indicating an absence of nephrotic range proteinuria. Cardiac disease was also unlikely as the transthoracic echocardiogram was within normal limits. Significant protein loss via the gastrointestinal tract contributed to the pleural effusions that required drainage of the left PleurX catheter three times a week. He also underwent right thoracentesis, and eventually right PleurX catheter placement during this hospitalization. Bilateral pleural studies were consistent with transudative effusions.

Total parenteral nutrition (TPN) was started as the patient was unable to tolerate a diet, with the addition of antiemetics and antidiarrheals for symptomatic relief. Intravenous methylprednisolone 250 mg was initiated for a total of three days and then the patient was transitioned to oral prednisone 80 mg daily, with gradual improvement in diarrhea. Protein supplements were added with eventual discontinuation of TPN. Prednisone was tapered down with the plan to taper further after discharge. However, tapering became challenging due to the worsening of diarrhea with a dose less than 20 mg daily. He was eventually started on sulfasalazine by his consultant rheumatologist with an improvement in symptoms and his fecal calprotectin decreased to 513 mcg/g four months later.

## Discussion

Our case represents the many challenges associated with the diagnosis and treatment of AIE. Current literature on this condition in adults is limited [2]. Clinicians should consider AIE in patients with chronic diarrhea and other coexisting autoimmune conditions such as hypothyroidism, diabetes mellitus, rheumatoid arthritis, and interstitial nephritis [1]. Although an association between coexisting autoimmune diseases and AIE has been established, the relationship between thymoma and AIE is not well understood [2,9]. A Mayo Clinic case series involved two patients, one with co-existing thymoma and another with post-thymoma resection [2]. Other than this study, only a handful of AIE cases have been linked to thymoma. Our case emphasizes the need to consider AIE as a sequela of thymoma resection [2]. Perhaps, upon the onset of gastrointestinal symptoms in patients who have undergone thymoma resection, evaluating for AIE should be considered to decrease the morbidity associated with the condition. In contrast, another question that should be considered is whether patients diagnosed with AIE should be evaluated for co-existing thymoma. Furthermore, the extent of protein loss was remarkable in our patient as it contributed to the recurrent pleural effusions, requiring invasive interventions. 

It is important to consider differential diagnoses including celiac disease and small intestinal bacterial overgrowth (SIBO) as villi blunting is also seen in these conditions [10]. In our patient, villous blunting was observed in both the duodenum and terminal ileum, which can be used to distinguish AIE from other conditions such as celiac disease and SIBO, where the duodenum is commonly affected [11]. Any aspect of the small intestine can be involved in AIE, but there is limited knowledge about the extent of duodenal, jejunal, or ileal involvement [12]. Capsule endoscopy may be useful in detecting gross mucosal changes [12].

Aggressive immunosuppression, most commonly with steroids, remains the mainstay treatment for AIE [1,7]. It was noted that 60% of patients responded to high-dose steroids; however, two-thirds of those patients became steroid-dependent or refractory, requiring additional immunomodulator therapy, such as azathioprine, 6-mercaptopurine, and infliximab, to maintain remission [7]. Similarly, in our patient, prednisone tapering resulted in worsening diarrhea, and thus, the initiation of an immunomodulator, sulfasalazine, was necessary. One study showed no improvement in AIE symptoms after radical thymectomy [13], whereas in another patient, thymectomy resulted in complete resolution of the gastrointestinal symptoms [14]. This highlights the variable nature of this condition and the need for further research on the relationship between AIE and thymomas. TPN is often required, as it was in our patient, due to the potential for malnutrition until symptoms are under control [7]. Additionally, our patient's fecal calprotectin level exceeded 5000 mcg/g, typically observed in inflammatory bowel diseases (IBD) [14]. While this marker reflects the severity of intestinal inflammation in IBD and is used for monitoring purposes [14], it may also be useful in AIE. As per our understanding, fecal calprotectin has not been commonly used in managing AIE. Nonetheless, our patient's fecal calprotectin level decreased significantly to 513 mcg/g as the symptoms improved over four months, suggesting its potential significance in disease monitoring.

## Conclusions

Considering AIE as a differential diagnosis when a patient presents with chronic diarrhea is crucial, especially in patients who have a history of thymoma or other autoimmune conditions. Our case study highlights the significant morbidity associated with the disease as well as the challenges in its management. Providers may consider calprotectin and the extent of small intestinal involvement as markers of disease severity. Because current knowledge is largely based on case reports, further research on AIE is important as its prevalence in adults is increasing.
